# Causal association between clonal hematopoiesis indeterminate potential and cancer: a Mendelian randomization study

**DOI:** 10.3389/ebm.2026.10923

**Published:** 2026-07-27

**Authors:** Juan Jin, Qian Pu, Chengrong Wu, Yu Lei, Yaxin Hu, Xiuju Yang, Jinghui Huang, Fangfang Wu, Li Xiao, Lei Yu

**Affiliations:** 1 Department of Obstetrics, The People’s Hospital of Qiannan, Duyun, Guizhou, China; 2 Central Laboratory, Guiyang Maternal and Child Health Care Hospital, Guiyang, Guizhou, China; 3 Reproductive Center, Guiyang Maternal and Child Health Care Hospital, Guiyang, Guizhou, China; 4 Prenatal Diagnosis Center, Affiliated Hospital of Guizhou Medical University, Guiyang, Guizhou, China; 5 Department of Obstetrics, The First People’s Hospital of Qingzhen, Qingzhen, Guizhou, China; 6 Prenatal Diagnosis Center, Qianxinan People’s Hospital, Xingyi, Guizhou, China

**Keywords:** cancer, clonal hematopoiesis of indeterminate potential, histological subtypes, Mendelian randomization, risk

## Abstract

Clonal hematopoiesis of indeterminate potential (CHIP) causes the expansion of mutated hematopoietic stem cells and produces immunophenotypically altered leukocytes, which induce a tumor-promoting inflammatory condition. However, the causal effect of CHIP on cancer remains unclear. We assessed the relationship of genetically predicted CHIP with the risk of 18 cancer types involving 612,576 cases using two-sample Mendelian randomization (MR). Genetic instruments for overall and sub-types of CHIP were obtained from the a large-scale genome-wide association study using independent (r^2^ < 0.001) SNPs at genome-wide significance (p < 5.0 × 10^-8^). Summary statistics for 18 cancers were obtained from the FinnGen, MVP, PLCO study, and large consortia. Inverse-variance weighted random-effects models were used as the primary method for estimating causal effects. Findings from independent datasets were combined using the fixed-effect model and Bonferroni corrections were applied for multiple testing. We found genetic predicted overall and DNMT3A CHIP was significantly associated with an increased risk of thyroid cancer, lung cancer, kidney cancer, brain cancer, basal cell carcinoma, and malignant melanoma after corrections. In addition, we found the causal estimate of CHIP varied across histological sub-types of cancer. Sensitivity analyses confirmed that these findings were robust. Strong associations were found between genetic predicted CHIP and an increased risk of a broad range of cancers, highlighting the importance of timely screening for CHIP in cancer early detection and prevention.

## Impact statement

Clonal hematopoiesis indeterminate potential (CHIP) defines the age-related expansion of hematopoietic clones harboring somatic mutations in the absence of overt hematologic abnormalities. Although CHIP has been associated with increased mortality in patients with solid cancer, the relationship between CHIP and solid tumor carcinogenesis remains largely unexplored. Considering the prominent role of inflammatory elements in the cancer microenvironment and emerging evidence positing the lung as a tumor of hematopoietic progenitor cells, CHIP may play a role in solid cancer pathogenesis. Indeed, CHIP is also a hot topic in cancer research recently. Using Mendelian randomization (MR) approach, this study found lifelong genetic propensity to develop overall CHIP and DNMT3A CHIP was significantly associated with increased risk of thyroid cancer, lung cancer, kidney cancer, brain cancer, basal cell carcinoma, and malignant melanoma. In addition, we found the causal estimate of CHIP vary across histological subtypes of cancer. Multiple sensitivity analyses and replication analyses also supported this observation. To our knowledge, this is the first comprehensive genetic instrumental variable study to depict the relationship between CHIP and solid cancer. Our study found significant association between genetic predicted CHIP and an increased risk of a broad range of cancers, highlighting the importance of timely screening for CHIP in cancer early detection and prevention.

## Introduction

Clonal hematopoiesis of indeterminate potential (CHIP) is an age-related expansion of hematopoietic stem cells (HSCs) harboring oncogenic somatic mutations (variant allele frequency, VAF >2%) in the absence of clinical hematologic disorders. Among the list of genes identified as potential drivers of CHIP, DNMT3A, TET2, and ASXL1 are the most frequently mutated [1, 2]. In addition to its potential as a risk factor in hematological cancer [2, 3], CHIP is also linked with various non-hematological conditions, such as cardiovascular disease [4, 5], pulmonary disease [6], liver disease [7], and neurological disease [8]. Although the biological mechanisms underlying these associations are largely unknown, accumulative evidence has indicated that CHIP can induce aberrant inflammatory responses or impair the immune function, which are considered as key pathogenic features contributing towards malignant transformation [9–11].

A number of cohort studies have reported the presence of CHIP in solid tumor samples from a treatment naïve setting, further confirming infiltration of the solid tumor microenvironment by clonal hematopoiesis mutated hematopoietic cells [12–14]. Considering the prominent role of chronic inflammation in cancer development [15, 16] and the pervasive nature of circulating immune cells, CHIP may play a key role in cancer pathogenesis. Further, previous clinical studies reported certain cancers are more likely than others to be associated with CHIP [12, 13, 17], suggesting that CHIP may have distinct effects on different cancers. However, the causality of CHIP and cancer can be difficult to establish since their relationship is confounded by factors such as aging and some anti-cancer treatments. Moreover, germline causes of CHIP have been recently identified [18–20], and the potential influence of genetic susceptibility to CHIP on cancer development remains poorly understood [21]. Therefore, a comprehensive appraisal of the causal associations between CHIP and cancer is of great importance in cancer prevention.

Mendelian randomization (MR) uses germline genetic variants as proxies (or instrumental variables) for risk factors to infer a causal relation between factors on disease outcomes and thus overcome some of the conventional issues (e.g., unmeasured confounding and reverse causation bias) in observational settings [22]. In addition, MR analysis evaluates the causality for lifelong effects of risk factors on disease outcomes, which is important in the context of diseases like cancer where a long induction and latent period may be required for a particular risk factor to generate effect.

To date, no studies have comprehensively evaluated the causal effects of CHIP across cancer. Given the emerging role of CHIP in cancer etiology, we used a two-sample MR framework to examine potential causal associations between genetically predicted CHIP and the risk of 18 types of cancer.

## Materials and methods

### Research design

**FIGURE 1:**
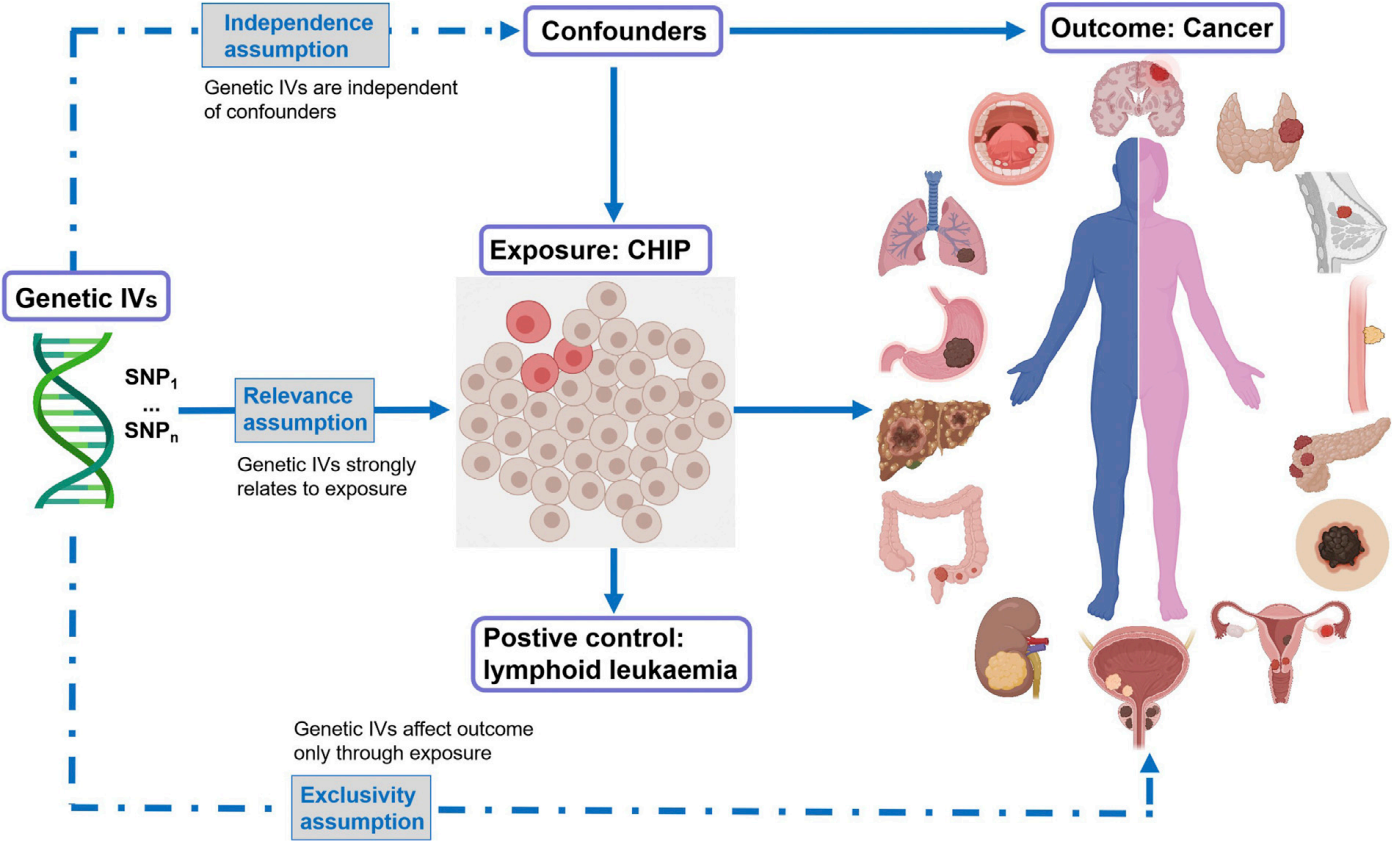
Study overview.

This study followed the Strengthening the Reporting of Observational Studies in Epidemiology-Mendelian Randomisation reporting guidelines (Supplementary Table S1) [23]. First, instruments to proxy CHIP exposure were generated and validated. Then, we used univariable two-sample MR to investigate the associations between genetically predicted CHIP and cancer risk. An outline of the current MR study can be found in Figure 1. All GWAS studies were approved by their respective ethical review committees and permitted the use of summary data without restriction; therefore, no further ethical approval or informed consent was required for the present study.

### Genetic instrument construction and validation

Summary data for overall CHIP [inclusive CHIP (with at least one CHIP mutation) and exclusive CHIP (without CHIP mutation)] and main CHIP sub-types (DNMT3A CHIP and TET2 CHIP) were obtained from the a large-scale Genome-Wide Association Study (GWAS) performed in European cohorts, adjusting for sex, age, and population structure [20]. Single nucleotide polymorphisms (SNPs) with minor allele frequency >0.01 associated with overall, DNMT3A, and TET2 CHIP at genome-wide significance (p < 5 × 10^−8^) were considered as genetic instruments. The SNPs were further clumped by the linkage disequilibrium (LD) threshold r^2^ 0.001 within 10,000 kilobases windows to ensure they were independent from each other. The strength of each genetic instrument was estimated using F-statistic; an F-statistic greater than 10 indicates minimally weak instrument bias [24]. As the presence of CHIP has been associated with an increased risk of hematological malignancies, we tested associations of our instrument for CHIP with lymphoid leukemia in positive control MR analysis.

### Cancer GWAS data sources

This study included 18 types of cancer outcomes: brain, oral, thyroid, esophageal, lung, breast, stomach, pancreatic, liver, colorectal, bladder, prostate, kidney, ovarian, endometrial, cervical, basal cell carcinoma, and malignant melanoma. We obtained summary genetic associations with these outcomes from the FinnGen consortium [25], Million Veteran Program (MVP) [26], PLCO atlas [27], large consortia (e.g., BCAC: The Breast Cancer Association Consortium; TRICL: The Transdisciplinary Research of Cancer in Lung), and other larger GWAS meta-analysis. When sample overlapping was identified, we used the summary statistics from the study with the largest sample size. All individuals involved in each cancer GWAS analysis were of European ancestry. Detailed information on the diagnostic criteria for cancer, genotyping method, imputation, and quality control measures for these GWAS is available in the original publications. The GWAS summary for lymphoid leukemia used in the positive control MR was obtained from the FinnGen consortium.

### Phenome-wide association analysis and pathway analysis

We performed SNP-level phenome-wide association studies (PheWAS) for the genetic instruments using Open Target Platform (https://genetics.opentargets.org/) [28] for secondary phenotypes associated with traditional cancer risk factors (P < 5 × 10^−8^) to further explore the impact of horizontal pleiotropy on causality estimates. Formal pathway enrichment analysis of the instrument-tagged gene set was performed using the WebGestalt online toolkit [29]. Over-representation analysis (ORA) was applied to identify significantly enriched biological pathways based on three canonical pathway databases: the Kyoto Encyclopedia of Genes and Genomes (KEGG), Reactome, and WikiPathways. All analyses were conducted for *Homo sapiens* with the gene symbol as the input identifier. Multiple testing bias was corrected for using the Benjamini-Hochberg false discovery rate (FDR) method, and pathways with an adjusted FDR value <0.05 were defined as statistically significant.

### Statistical analysis

When estimating the causal effects of CHIP exposure on cancer outcome, we used the random-effects inverse-variance weighted (IVW) approach as the primary method [30]. Multiple sensitivity analyses, which each make different assumptions of validity of the genetic instruments including the weighted median, MR-Egger, weighted mode, simple mode, and MR-RAPS (Robust Adjusted Profile Score) [31], were performed to examine the robustness of the results. The MR-PRESSO (Pleiotropy RESidual Sum and Outlier) test was also performed to detect horizontal pleiotropy (global test) and correct the effect of outliers [32]. The heterogeneity of genetic variants was examined using Cochran Q-statistic, whereas pleiotropy between genetic variants was evaluated by the MR-Egger intercept test [33]. In addition, iterative leave-one-out analysis was conducted by removing one SNP at a time from instruments to assess the influence of individual variants. The MR Steiger test was also performed to confirm the direction of causal estimation [34]. Furthermore, we examined the effects of genetic susceptibility to cancer on CHIP in reverse MR, wherein uncorrelated (r^2^ < 0.001) SNPs associated with specific types of cancer at a genome-wide significance (p < 5 × 10^−8^) were selected as genetic instruments. The multivariable MR (MVMR) analysis was performed to identify the independent risk factors (e.g., telomere length and smoking). Moreover, the odds ratios (ORs) with a 95% confidence interval (CI) was scaled to per standard deviation (SD) increase in genetically predicted overall or sub-types of CHIP. When the associations were generated from multiple datasets for the same cancer outcome, the combined effects were obtained using the fixed-effect meta approach. The online tool mRnd was used to calculate statistical power (http://cnsgenomics.com/shiny/mRnd/). The association with a nominal p value <0.05 but a Bonferroni adjusted p value >6.94 × 10^-4^(0.05 Bonferroni-corrected for four types of CHIP with 18 cancer outcomes) was considered suggestive, and the association with a Bonferroni adjusted p value <6.94 × 10^−4^ was deemed significant. All analyses were two-sided and were conducted using the “TwoSampleMR” (V.0.5.7), “MendelianRandomization” (0.7.0), and “meta” (V.6.5-0) packages in R (version 4.3.0).

## Results

### Genetic variant selection

In total, 26 SNPs were used to proxy overall inclusive CHIP; 21 SNPs proxied overall exclusive CHIP; 26 SNPs proxied DNMT3A sub-type; and six SNPs proxied TET2 sub-type. For CHIP traits, the genetic instruments used explained approximately 0.52%, 0.44%, 0.56%, and 0.1% of variance in overall inclusive CHIP, overall exclusive CHIP, DNMT3A sub-type, and TET2 sub-type, respectively. Characteristics of genetic variants used to instrument CHIP are presented in Supplementary Table S2. The F-statistics of selected genetic instruments for CHIP ranged from 30.0 to 507.5, indicating minimally weak instrument bias. The positive control analyses identified associations in the expected direction between genetically predicted CHIP and lymphoid leukemia (Supplementary Table S3), further confirming the validity of the genetic instruments.

### Main results

**FIGURE 2:**
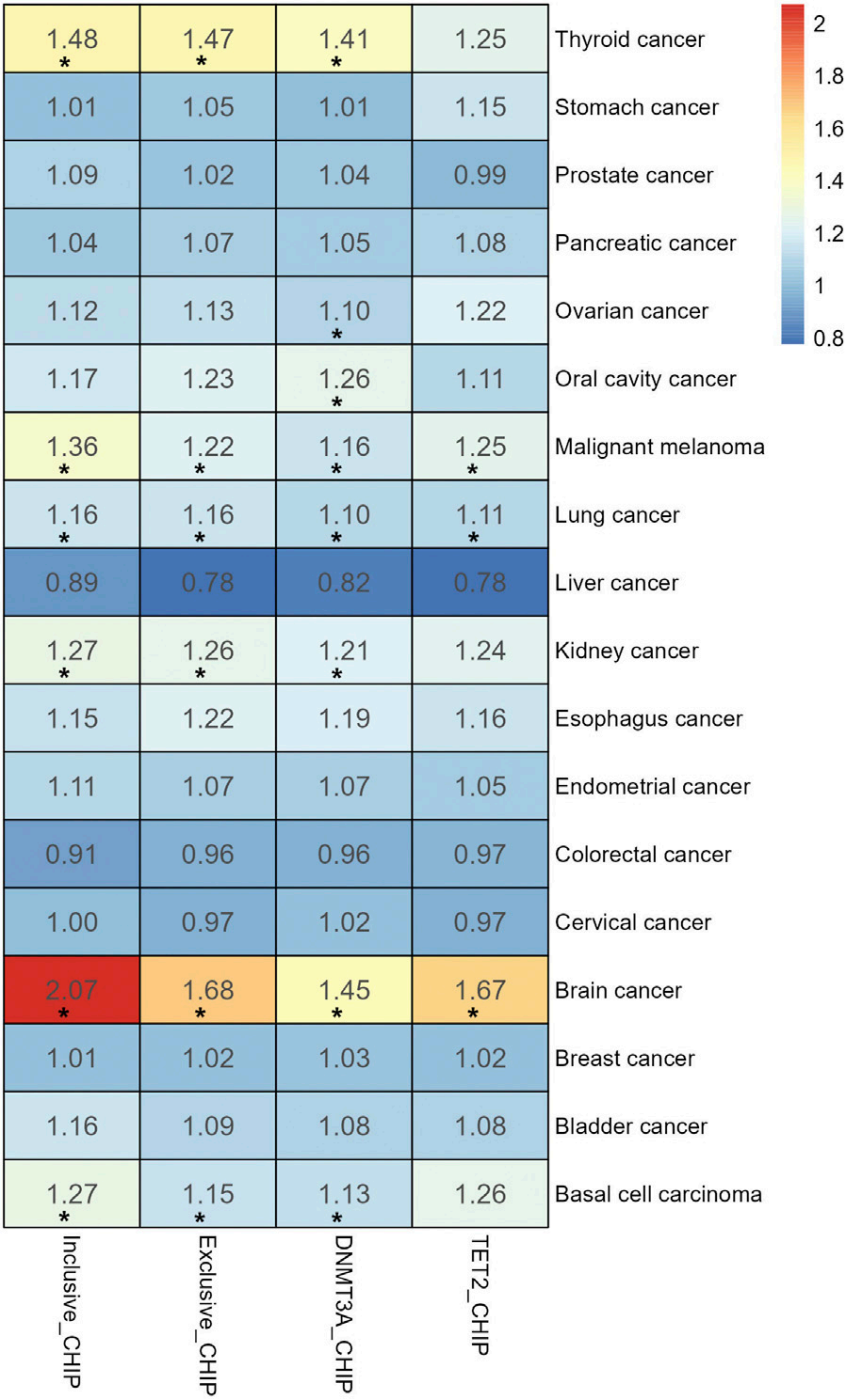
Summary of associations of genetically predicted overall and subtypes of CHIP with cancer. Numbers in the boxes are ORs for associations of exposure and each cancer outcome. The reported ORs are scaled to a 1-SD increase in log odds of genetic susceptibility to overall or subtypes of CHIP. *p < 0.05 after multiple testing correction.

The characteristics of the cancer outcome and cancer histological sub-types GWAS data involving a total of 612,576 cancer cases are summarized in Supplementary Table S4. We had 80% power to detect an association with an OR of 1.5 or more at an α of 0.05 for most cancer outcomes (Supplementary Table S5). Among the 18 types of cancer included in analyses, we found consistent evidence for the association of CHIP in the risk of six site-specific cancers (brain cancer, thyroid cancer, kidney cancer, lung cancer, basal cell carcinoma, and malignant melanoma) (Figure 2). However, we found limited evidence that genetically predicted overall CHIP or its subtypes were causally associated with the risk of breast cancer, oral cavity cancer, esophageal cancer, stomach cancer, pancreatic cancer, liver cancer, colorectal cancer, bladder cancer, prostate cancer, endometrial cancer, ovarian cancer, and cervical cancer (Supplementary Table S6). Results from multiple MR method analyses support these associations (Supplementary Table S7).

### Brain cancer

**FIGURE 3:**
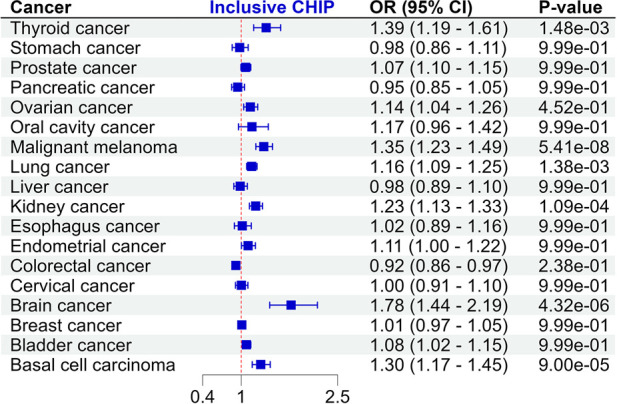
Associations of genetic susceptibility to inclusive CHIP with cancer. Mendelian randomization estimates were generated using the IVW approach. P values are for ORs (95% CIs) after multiple testing correction.

**FIGURE 4:**
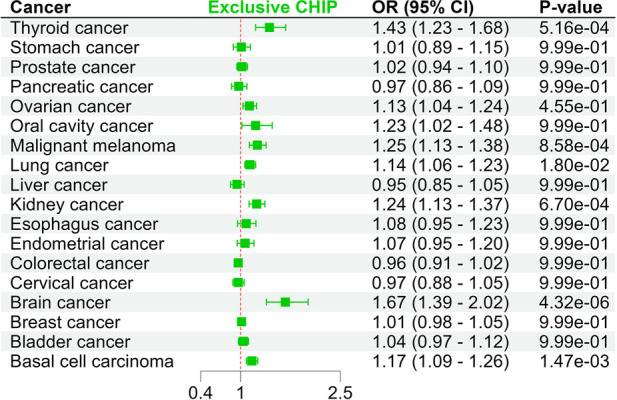
Associations of genetic susceptibility to exclusive CHIP with cancer. Mendelian randomization estimates were generated using the IVW approach. P values are for ORs (95% CIs) after multiple testing correction.

**FIGURE 5:**
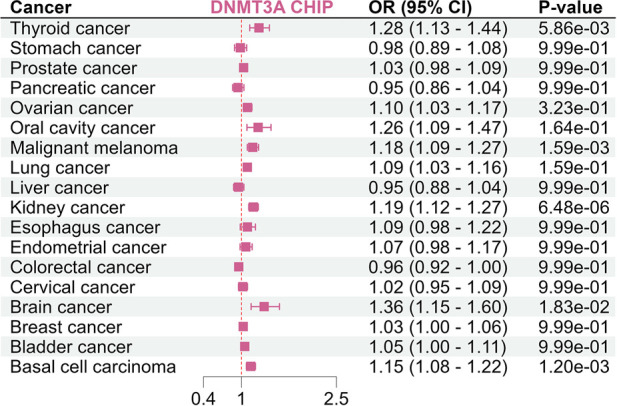
Associations of genetic susceptibility to DNMT3A CHIP with cancer. Mendelian randomization estimates were generated using the IVW approach. P values are for ORs (95% CIs) after multiple testing correction.

**FIGURE 6:**
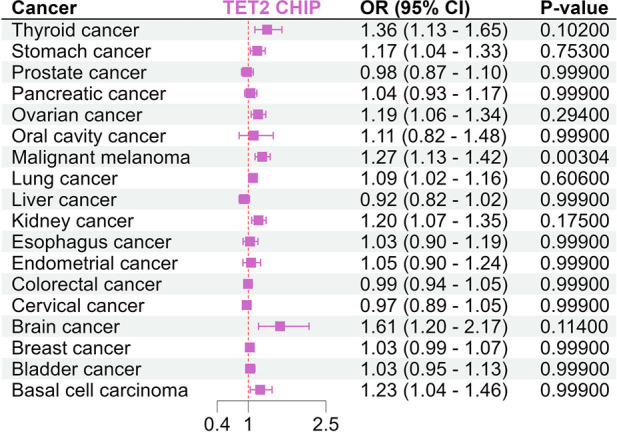
Associations of genetic susceptibility to TET2 CHIP with cancer. Mendelian randomization estimates were generated using the IVW approach. P values are for ORs (95% CIs) after multiple testing correction.

We observed a strong causal association between overall and sub-types of CHIP and brain cancer in both FinnGen and MVP datasets. The pooled OR of brain cancer was 1.78 (95% CI: 1.44–2.19, p = 4.32 × 10^−6^, Figure 3) for over inclusive CHIP, 1.67 (95% CI: 1.39–2.02, p = 4.32 × 10^−6^, Figure 4) for over exclusive CHIP, 1.36 (95% CI: 1.15–1.60, p = 0.02, Figure 5) for DNMT3A sub-type, and 1.61 (95% CI: 1.20–2.17, p = 0.11, Figure 6) for TET2 sub-type (Supplementary Table S6). The association for overall exclusive CHIP sustained in multivariable MR with adjustment for genetically predicted telomere length (OR = 1.35, 95% CI = 1.06–1.73, p = 0.017).

### Thyroid cancer

Across three independent datasets involving 5034 cases, there was consistent evidence of an association between overall inclusive CHIP and increased risk of thyroid cancer with a pool OR of 1.39 (95% CI: 1.19–1.61, p = 1.48 × 10^−3^). Significant associations were also observed for overall exclusive CHIP (OR: 1.43, 95% CI: 1.22–1.68, p = 5.15 × 10^−4^) and DNMT3A sub-type after Bonferroni correction (OR: 1.28, 95% CI: 1.13–1.44, p = 5.86 × 10^−3^, Figure 5). The association of overall exclusive CHIP was maintained in multivariable MR with adjustment for genetically predicted telomere length (OR = 1.24, 95% CI = 1.06–1.45, p = 7.56 × 10^−3^).

Histological sub-types of thyroid cancer analysis using data obtained from the FinnGen consortium found strong evidence for an association of genetically predicted overall CHIP (inclusive CHIP, OR: 1.40, 95% CI: 1.13–1.74, p = 0.002; exclusive CHIP, OR: 1.33, 95% CI: 1.06–1.66, p = 0.01) and DNMT3A sub-type (OR: 1.23, 95% CI: 1.01–1.49, p = 0.04) with papillary adenocarcinoma risk (Supplementary Table S8).

### Lung cancer

Across four independent datasets involving 44,784 cases, we identified associations in a consistent direction between CHIP and lung cancer. Genetically predicted overall inclusive CHIP was strongly associated with an increased risk of lung cancer with a pooled OR of 1.16 (95%CI: 1.08–1.25, p = 1.38 × 10^−3^). A similar finding was also observed for overall exclusive CHIP (OR: 1.14, 95% CI: 1.06–1.23, p = 0.02). Similar results by sub-types of CHIP were also found for lung cancer (DNMT3A CHIP, OR: 1.10, 95%CI: 1.04–1.17, p = 0.009; TET2 CHIP, OR: 1.11, 95%CI: 1.04–1.17, p = 0.02). In addition, results from multivariable MR analysis confirmed that overall exclusive CHIP (OR = 1.16, 95% CI = 1.05–1.28, p = 4.86 × 10^−3^) was an independent risk factor of lung cancer after adjusting for lifetime smoking index. However, neither the genetically predicted DNMT3A subtype nor TET2 subtype showed any association with lung cancer after multiple testing corrections.

We then assessed associations between CHIP and the risk of three histological sub-types of lung cancer, namely squamous cell lung cancer, lung adenocarcinoma, and small cell lung carcinoma. The IVW estimates for having inclusive, exclusive, and DNMT3A CHIP with incident lung adenocarcinoma risk were 1.48 (95% CI: 1.15–1.90, p = 2.09 × 10^-3^), 1.44 (95% CI: 1.11–1.85, p = 5.27 × 10^-3^), and 1.25 (95% CI: 1.04–1.50, p = 0.01), respectively. However, we did not identify any genetic evidence that CHIP may increase the risk of squamous cell lung cancer or small cell lung carcinoma (Supplementary Table S9).

### Kidney cancer

Significant associations between genetically predicted overall CHIP (inclusive CHIP, OR: 1.22, 95% CI: 1.13–1.33, p = 1.09 × 10^−4^; exclusive CHIP, OR: 1.24, 95% CI: 1.13–1.36, p = 6.70 × 10^−4^) and DNMT3A CHIP (OR: 1.19, 95% CI: 1.11–1.26, p = 6.48 × 10^−6^) and an increased risk of kidney cancer were identified from pooled analyses. Likewise, our multivariable MR analysis showed a robustness of associations for genetically predicted telomere length (Supplementary Table S2).

When stratified by histological sub-types, an adverse effect of CHIP appeared to be mainly associated with clear cell renal cell carcinoma (inclusive CHIP, OR: 1.31, 95% CI: 1.16–1.49, p = 2.21 × 10^−5^; exclusive CHIP, OR: 1.27, 95% CI: 1.10–1.47, p = 9.09 × 10^−4^; DNMT3A CHIP, OR: 1.23, 95% CI: 1.13–1.34, p = 1.07 × 10^−6^), with limited evidence in papillary renal cell carcinoma (inclusive CHIP, OR: 1.25, 95% CI: 0.98–1.58; exclusive CHIP, OR: 1.18, 95% CI: 0.92–1.51; DNMT3A CHIP, OR: 1.16, 95% CI: 0.96–1.39, Supplementary Table S10).

### Basal cell carcinoma

For a 1-unit increase in log-transformed odds of CHIP, the combined OR for basal cell carcinoma estimated from FinnGen and MVP was 1.30 (95% CI: 1.17–1.45, p = 9.0 × 10^−5^) for overall inclusive CHIP, 1.17 (95% CI: 1.09–1.26, p = 1.47 × 10^−3^) for overall exclusive CHIP, and 1.15 (95% CI: 1.08–1.22, p = 1.20 × 10^−3^) for DNMT3A CHIP. When restricting multivariable MR analysis corrected for telomere length, we obtained similar results, with overall exclusive CHIP remaining associated with basal cell carcinoma (Supplementary Table S19).

### Malignant melanoma

In the combined datasets, our MR analysis discovered that genetically predicted CHIP or its sub-types could increase the risk of malignant melanoma after Bonferroni adjustment, which was supported by different MR methods (Supplementary Table S7). As estimated by the IVW method, the odds of malignant melanoma was 1.35 (95% CI: 1.23–1.49, p = 5.41 × 10^−8^) for inclusive CHIP, 1.25 (95% CI: 1.13–1.38, p = 8.57 × 10^−4^) for exclusive CHIP, 1.18 (95% CI: 1.09–1.27, p = 0.002) for DNMT3A CHIP, and 1.25 (95% CI: 1.09–1.43, p = 0.003) for TET2 CHIP. Multivariable MR adjusting for telomere length provided strong evidence for genetically proxied overall exclusive CHIP on higher malignant melanoma risk (Supplementary Table S19).

### Sensitivity analysis

Results for genetic susceptibility to CHIP and risk of cancer outcomes were directionally consistent in alternative MR methods (Supplementary Table S7). The MR pleiotropy residual sum and outlier test identified one to seven outliers, but similar magnitude associations were observed after removal of these variants (Supplementary Table S13). Although some heterogeneity was detected, it did not invalidate the IVW using a random effects model of MR results, which may balance the combined heterogeneity (Supplementary Table S14). No bidirectional effects were identified using the MR-Steiger directionality test (Supplementary Table S15). In reverse MR analyses, genetic susceptibility to cancer was not significantly associated with CHIP or its sub-types (Supplementary Table S16). In addition, no horizontal pleiotropy was observed in MR-Egger intercept analysis (Supplementary Table S17). After examining the Open Targets Platform, we found that several of the CHIP genetic variants were also associated with blood cell-related phenotypes and telomere length, which may result in the presence of horizontal pleiotropy (Supplementary Table S18). We then repeated the primary analyses using a genetic instrument excluding the TERT (rs7705526 and rs2853677) and the TP53 locus (rs78378222), but similar magnitude associations were observed when these variants were excluded from the analyses (Supplementary Table S20). Furthermore, pathway enrichment analysis suggested that that instrument-tagged genes were significantly enriched in drug resistance, DNA damage and hematopoietic regulatory pathways (Supplementary Figure 1). Moreover, the results from the leave-one-out sensitivity analysis did not reveal any influential SNPs driving the associations (Supplementary Figures S2–S19).

## Discussion

We systematically assessed the causal association of genetic susceptibility to overall and sub-types of CHIP with the risk of 18 cancer types. Genetic instruments for CHIP were obtained from a large-scale GWAS, which improved the statistic power for the MR analysis. In line with findings from previous studies [19, 20], we found that genetic susceptibility to CHIP was associated with an increased risk of lung cancer, malignant melanoma, and basal cell carcinoma. We identified several novel associations of genetic susceptibility to CHIP with an increased risk of thyroid cancer, kidney cancer, and brain cancer. We also found significant associations of genetically predicted CHIP with some histological subtypes of cancer (e.g., papillary adenocarcinoma of the thyroid and clear cell renal cell carcinoma).

The clinical relevance of CHIP to thyroid cancer has been reported by several observational studies. Coombs et al. showed that thyroid carcinoma was the most common solid tumor associated with CHIP, with a prevalence of 36% [35]. Similarly, a recent study found that the prevalence of CHIP in patients with thyroid cancer was 37% [36]. However, the causal effect of CHIP on thyroid cancer remains unclear. It has been proposed that radioactive iodine (RAI) exposure may induce the high prevalence of CHIP in patients with thyroid cancer, whereas previous findings exploring the effect of RAI on CHIP among thyroid cancer patients were inconsistent [36, 37]. Although the detailed mechanism by which the hematopoietic system interacts with thyroid cancer cells remains unknown, genetic evidence from our MR study suggested that CHIP might increase the risk of thyroid cancer in the long-term. Since genetic predicted over exclusive CHIP was also found to be associated with thyroid cancer, mechanisms other than RAI may also be at play, such as tumor-associated macrophages found in anaplastic thyroid cancer [38].

We found strong evidence for an association between genetically predicted CHIP and kidney cancer risk. Although prospective studies investigating associations of CHIP with the development of kidney cancer are limited, intriguing findings have been reported. CHIP was significantly associated with various adverse kidney outcomes including acute kidney injury [10], chronic kidney disease [39], and kidney function decline [40]. Subsequent evidence from animal studies also supported the causal effects of CHIP on kidney inflammation and fibrosis [10, 41]. Given the positive feedback loop between CHIP and inflammation, a possible mechanism explaining the relation between kidney cancer and CHIP may be the clonal expansion in both myeloid and lymphoid cell lines. However, the mechanisms driving the association identified in our MR analysis should be further elucidated in future studies.

There was strong evidence to support a positive association of genetically predicted overall and sub-types of CHIP with brain cancer. There is low possibility of false-positive and reverse causation in our analysis because of the strict genetic instruments selection procedure and MR Steiger test, alongside the consistent findings observed from two independent studies, which further improve the robustness of the MR analysis. Although epidemiological evidence for the association of CHIP in brain cancer is scarce, large longitudinal studies may support the benefit of monitoring healthy people with CHIP to optimize disease prediction models [42].

Previous studies suggested that CHIP could contribute to the development of prostate cancer, breast cancer, and ovarian cancer [18, 19]. However, other studies reported null or inverse associations [43, 44]. Our MR results found genetically predicted CHIP was not causally associated with prostate cancer, breast cancer, or ovarian cancer, and we deemed that the previously observed association might be confounded by cancer treatments (e.g., chemotherapy), limited sample sizes, and sampling variability. In addition, the causal effect of CHIP may differ depending on the tissue micro-environment and immune interactions [45, 46]. Indeed, a recent large-scale cohort study found that primary tumor regions from patients with CHIP had a higher representation of myeloid cells compared to tumor regions from patients without CHIP [47]. However, the micro-environment or immune interactions could not be assessed on the basis of the summary-level data. Consequently, the putative influence of CHIP in the development of these cancers remains uncertain and further investigations are needed to confirm our findings.

PheWAS and pathway enrichment analysis suggested that several mechanistically distinct classes of genetic loci were associated with CHIP: loci conferring CHIP-intrinsic, cell-autonomous clonal selection mechanisms, and proxy loci tagging upstream systemic hematopoietic regulatory processes that independently promote both hematopoietic clonal expansion and malignant transformation [1, 2]. Indeed, several enriched pathways directly align with core CHIP pathogenesis, including somatic mutation surveillance, hematopoietic stem cell (HSC) clonal competition, and DNA damage response signaling [48]. These mechanisms act in a CHIP-specific manner, driving the selective outgrowth of somatically mutated HSC clones and defining the initiation and progression of CHIP itself. In contrast, multiple top-enriched pathways represent upstream foundational biological regulators, namely telomere homeostasis, HSC proliferative control, and B-cell developmental biology, which serve as shared predisposing determinants rather than CHIP-exclusive drivers [49]. Genetically regulated telomere length shapes lifelong HSC replicative reserve and genomic stability; shortened telomeres increase replicative stress, facilitate clonal selection, and elevate cancer risk through genome instability, independent of CHIP somatic mutations [50, 51]. Similarly, altered HSC proliferation programs expand the vulnerable HSC pool, increasing the likelihood of somatic mutation accumulation and clonal dominance while promoting oncogenic transformation via cell-cycle dysregulation. Enriched B-cell biology pathways further reflect adaptive immune microenvironment modulation, which indirectly remodels bone marrow niche conditions to favor clonal expansion and subsequent malignancy [52]. It is possible that CHIP-specific loci directly mediate clonal hematopoiesis initiation, whereas telomere, HSC proliferation, and B-cell-related loci represent upstream pleiotropic factors that independently cause both age-related clonal expansion and cancer predisposition through distinct, CHIP-independent biological pathways.

Strengths of this analysis include the systematic evaluation of CHIP, representative of overall and sub-types of CHIP, in the risk of 18 cancer types using large-scale GWAS data from different resources, which ensured adequate statistical power and the robustness of findings. Because of different study designs and powers of different datasets, definitive conclusions are hard to draw. However, there is little possibility of false-positive and reverse causation in our study because of the application of strict instrument selection, validation processes, and the MR Steiger test as well as the consistent results in FinnGen, MVP, and PLCO studies. Large consortia and other larger GWAS meta-analyses also supported these findings. Additional support for the robustness of our findings comes from the directionally consistent estimates across MR methods (e.g., IVW, weighted median, and MR-RAPS) and the absence of horizontal pleiotropy (MR‐Egger intercepts and MR‐PRESSO estimations). Results from Open Target Platform searching also confirmed that none of the selected genetic variants were deemed to be at risk of horizontal pleiotropy.

Despite these strengths, potential limitations should be considered. First, our MR analysis estimated the effect of CHIP based on germline predictors of CHIP rather than the direct effects of CHIP. This is based on the assumption that the effect of genetically predicted CHIP is proportional to its overall impact, which may not be in line with its actual mechanism. Second, statistical power can be limited in some specific cancers (e.g., brain cancer) and histological subtypes (e.g., follicular thyroid cancer or clear cell ovarian cancer) due to small sample sizes. It is still essential to confirm our results through larger sample sizes. Third, the gene-gene or gene-environment interactions and nonlinearity effects of CHIP on cancer could not be estimated using summary-level data. Fourth, we cannot definitively rule out the possibility of horizontal pleiotropy, although we performed various sensitivity analyses and showed the validity of these genetic instruments. For example, CHIP with TET2 mutations has been linked to cancer-associated risk factors including obesity [53] and systemic inflammation [5]. Fifth, horizontal pleiotropy cannot be excluded when using SNPs located at TERT and TP53 regions as instruments for CHIP in primary analysis. We conducted additional sensitivity analyses using genetic instruments excluding the TERT and the TP53 locus for CHIP to test for the influence of pleiotropy on our estimates, and our results remain robust. Additionally, our analyses did not adjust for chemotherapeutic treatment or blood sampling time of cancer cases, which may influence causal estimates. Lastly, our study comprised only European cohorts, which limits the generalizability of the findings to other ethnic populations.

In conclusion, our study demonstrates that genetic susceptibility to CHIP is related to an increased risk for thyroid cancer, lung cancer, kidney cancer, brain cancer, basal cell carcinoma, and malignant melanoma. Our findings provide genetic support that CHIP may serve as a novel biomarker for early cancer detection and highlight the importance of timely screening for CHIP in cancer prevention.

## Data Availability

Data are available in a public, open access repository. The summary statistics of FinnGen GWAS data can be accessed at https://www.finngen.fi/en. The PLCO GWAS summary statistics can be accessed at https://exploregwas.cancer.gov/plco-atlas/). The summary level statistics from UKBB GWAS can be accessed via https://www.nealelab.is/uk-biobank. Summary statistics of cancer and CHIP used in this study are publicly available on the GWAS Catalog project (https://www.ebi.ac.uk/gwas/).
