# Supplementary material for: Causal association between clonal hematopoiesis indeterminate potential and cancer: a Mendelian randomization study
Source: Exp Biol Med (Maywood). 2026 Jul 27;251:10923. doi: 10.3389/ebm.2026.10923 (PMC13455644; doi:10.3389/ebm.2026.10923)
Supplement: Supplementary file 1 [file Supplementaryfile1.docx]

**Supplementary Figure 1**. Pathway enrichment of the gene set tagged by the genetic instruments.

**Supplementary Figure 2**. Forest plot for leave-one-out analysis of brain cancer, with each point denoting the causal effect by IVW after removing the specific SNP. (A) analysis based on MVP GWAS, (B) analysis based on FinnGen GWAS.

(A) MVP GWAS

(B) FinnGen GWAS

**Supplementary Figure 3**. Forest plot for leave-one-out analysis of oral cavity cancer, with each point denoting the causal effect by IVW after removing the specific SNP. (A) analysis based on FinnGen GWAS, (B) analysis based on oral cancer GWAS.

(A) FinnGen GWAS

(B) Oral cavity cancer GWAS

**Supplementary Figure 4**. Forest plot for leave-one-out analysis of thyroid cancer, with each point denoting the causal effect by IVW after removing the specific SNP. (A) analysis based on MVP GWAS, (B) analysis based on FinnGen GWAS, (C) analysis based on PLCO GWAS.

(A) MVP GWAS

(B) FinnGen GWAS

(C) PLCO GWAS

**Supplementary Figure 5**. Forest plot for leave-one-out analysis of esophageal cancer, with each point denoting the causal effect by IVW after removing the specific SNP. (A) analysis based on MVP GWAS, (B) analysis based on FinnGen GWAS, (C) analysis based on PLCO GWAS.

(A) MVP GWAS

(B) FinnGen GWAS

(C) PLCO GWAS

**Supplementary Figure 6**. Forest plot for leave-one-out analysis of lung cancer, with each point denoting the causal effect by IVW after removing the specific SNP. (A) analysis based on MVP GWAS, (B) analysis based on FinnGen GWAS, (C) analysis based on PLCO GWAS, (D) analysis based on lung cancer TRICL GWAS.

(A) MVP GWAS

(B) FinnGen GWAS

(C) PLCO GWAS

(D) Lung cancer TRICL GWAS

**Supplementary Figure 7**. Forest plot for leave-one-out analysis of breast cancer, with each point denoting the causal effect by IVW after removing the specific SNP. (A) analysis based on MVP GWAS, (B) analysis based on FinnGen GWAS, (C) analysis based on PLCO GWAS (not avaiable for TET2 CHIP due to insufficient SNP), (D) analysis based on breast cancer BCAC GWAS.

(A) MVP GWAS

(B) FinnGen GWAS

(C) PLCO GWAS

(D) Breast cancer BCAC GWAS

**Supplementary Figure 8**. Forest plot for leave-one-out analysis of stomach cancer, with each point denoting the causal effect by IVW after removing the specific SNP. (A) analysis based on MVP GWAS, (B) analysis based on FinnGen GWAS, (C) analysis based on PLCO GWAS.

(A) MVP GWAS

(B) FinnGen GWAS

(C) PLCO GWAS

**Supplementary Figure 9**. Forest plot for leave-one-out analysis of pancreatic cancer, with each point denoting the causal effect by IVW after removing the specific SNP. (A) analysis based on MVP GWAS, (B) analysis based on FinnGen GWAS, (C) analysis based on PLCO GWAS.

(A) MVP GWAS

(B) FinnGen GWAS

(C) PLCO GWAS

**Supplementary Figure 10**. Forest plot for leave-one-out analysis of liver cancer, with each point denoting the causal effect by IVW after removing the specific SNP. (A) analysis based on MVP GWAS, (B) analysis based on FinnGen GWAS, (C) analysis based on PLCO GWAS.

(A) MVP GWAS

(B) FinnGen GWAS

(C) PLCO GWAS

**Supplementary Figure 11**. Forest plot for leave-one-out analysis of colorectal cancer, with each point denoting the causal effect by IVW after removing the specific SNP. (A) analysis based on MVP GWAS, (B) analysis based on PLCO GWAS, (C) analysis based on colorectal cancer GWAS.

(A) MVP GWAS

(B) PLCO GWAS

(C) Colorectal cancer GWAS

**Supplementary Figure 12**. Forest plot for leave-one-out analysis of bladder cancer, with each point denoting the causal effect by IVW after removing the specific SNP. (A) analysis based on MVP GWAS, (B) analysis based on FinnGen GWAS, (C) analysis based on PLCO GWAS.

(A) MVP GWAS

(B) FinnGen GWAS

(C) PLCO GWAS

**Supplementary Figure 13**. Forest plot for leave-one-out analysis of prostate cancer, with each point denoting the causal effect by IVW after removing the specific SNP. (A) analysis based on MVP GWAS, (B) analysis based on FinnGen GWAS, (C) analysis based on PLCO GWAS, (D) analysis based on prostate cancer PRACTICAL GWAS.

(A) MVP GWAS

(B) FinnGen GWAS

(C) PLCO GWAS

(D) Prostate cancer PRACTICAL GWAS

**Supplementary Figure 14**. Forest plot for leave-one-out analysis of kidney cancer, with each point denoting the causal effect by IVW after removing the specific SNP. (A) analysis based on MVP GWAS, (B) analysis based on PLCO GWAS, (C) analysis based on kidney cancer GWAS.

(A) MVP GWAS

(B) PLCO GWAS

(C) Kidney cancer GWAS

**Supplementary Figure 15**. Forest plot for leave-one-out analysis of ovarian cancer, with each point denoting the causal effect by IVW after removing the specific SNP. (A) analysis based on FinnGen GWAS, (B) analysis based on PLCO GWAS, (C) analysis based on ovarian cancer OCAC GWAS.

(A) FinnGen GWAS

(B) PLCO GWAS

(C) Ovarian cancer OCAC GWAS

**Supplementary Figure 16**. Forest plot for leave-one-out analysis of endometrial cancer, with each point denoting the causal effect by IVW after removing the specific SNP. (A) analysis based on PLCO GWAS, (B) analysis based on endometrial cancer GWAS.

(A) PLCO GWAS

(B) Endometrial cancer GWAS

**Supplementary Figure 17**. Forest plot for leave-one-out analysis of cervical cancer, with each point denoting the causal effect by IVW after removing the specific SNP.

**Supplementary Figure 18**. Forest plot for leave-one-out analysis of basal cell carcinoma, with each point denoting the causal effect by IVW after removing the specific SNP. (A) analysis based on MVP GWAS, (B) analysis based on FinnGen GWAS.

(A) MVP GWAS

(B) FinnGen GWAS

**Supplementary Figure 19**. Forest plot for leave-one-out analysis of malignant melanoma, with each point denoting the causal effect by IVW after removing the specific SNP. (A) analysis based on MVP GWAS, (B) analysis based on FinnGen GWAS, (C) analysis based on PLCO GWAS.

(A) MVP GWAS

(B) FinnGen GWAS

(C) PLCO GWAS
